# Springback Analysis for Warm Bending of Titanium Tube Based on Coupled Thermal-Mechanical Simulation

**DOI:** 10.3390/ma14175044

**Published:** 2021-09-03

**Authors:** Guangjun Li, Zirui He, Jun Ma, Heng Yang, Heng Li

**Affiliations:** 1 Chengdu Aircraft Industry (Group) Co., Ltd., Chengdu 610092, China; hzrjasmine@126.com; 2 State Key Laboratory of Solidification Processing, School of Materials Science and Engineering, Northwestern Polytechnical University, Xi’an 710072, China; jun.ma@ntnu.no (J.M.); yang_heng@mail.nwpu.edu.cn (H.Y.)

**Keywords:** titanium tube, warm bending, springback, coupled thermal-mechanical modeling

## Abstract

Titanium bent tubular parts attract extensive applications, thus meeting the ever-growing demands for light weight, high reliability, and long service life, etc. To improve bending limit and forming quality, local-heat-assisted bending has been developed. However, significant springback seriously reduces the dimensional accuracy of the bent tubular parts even under elevated forming temperatures, and coupled thermal-mechanical working conditions make springback behavior more complex and difficult to control in warm bending of titanium tubular materials. In this paper, using warm bending of thin-walled commercial pure titanium tube as a case, a coupled thermal-mechanical finite element model of through-process heating-bending-unloading is constructed and verified, for predicting the springback behavior in warm bending. Based on the model, the time-dependent evolutions of springback angle and residual stress distribution during thermal-mechanical unloading are studied. In addition, the influences of forming temperature and bending angle on springback angle, thickness variation, and cross-section flattening of bent tubes are clarified. This research provides a fundamental understanding of the thermal-mechanical-affected springback behavior upon local-heat-assisted bending for improving the forming accuracy of titanium bent tubular parts and structures.

## 1. Introduction

Titanium bent tubular components are widely used in high-end manufacturing industries, such as aircraft, aerospace, nuclear energy, etc., due to their high specific strength and excellent resistance to corrosion and fatigue [1,2,3]. With the limited formability of thin-walled titanium tubes under cold bending, a local-heat-assisted bending technology has been recently applied to achieve the bent tubular parts with a tighter bending radius and higher forming quality [4,5,6]. In the tube bending processes, however, one of the most important problems affecting dimensional accuracy of the formed parts is springback during unloading. It refers to the elastically-driven shape changes that happen when the external loads, such as tools, thermal/electric/magnetic fields, etc., are removed from the deformed part, which directly causes problems such as increased tolerances and variability in the subsequent forming operations, and problems in assembly and in the service of parts [7,8]. Therefore, accurate control of springback is of great importance to the fabrication of high-quality parts, which in turn requires a clear understanding of springback characteristics in the forming process.

Warm forming has been reported as a possible approach to reduce springback for some alloys. However, for titanium and its alloys, the ratio of yield stress to elastic modulus is still at a very high level even at elevated temperatures, resulting in a remarkable springback during warm forming [9,10]. From the view of time series, unloading is the final step in a forming operation so that springback is affected by thermal-mechanical history and presents a pronounced sensitivity to the variables involved in the through-process of both loading (forming) and unloading. In addition, compared with cold forming, the springback behavior in heat-assisted deformation is more complex due to the thermal effect on material properties, contact friction, thermal transferring, etc. [10,11,12]. All of these issues make springback in warm bending of titanium tubular materials a complicated problem and difficult to analyze and predict.

Up to now, there is plenty of research on the springback in tube and sheet forming by using analytical, numerical, and experimental methods. To name a few, Li et al. established an analytical-numerical hybrid modeling framework to clarify the neutral layer shifting phenomena and its underlying mechanisms in tube bending, and pointed out that the neutral layer can be positively controlled by heat-assisted bending [5,13]. Zhan et al. derived an analytical model for springback prediction in titanium tube bending, in which the influences of the initial tube specification and material properties on springback are obtained [14,15]. Li et al. studied the springback behaviors of the high strength titanium tube under multi-die constrained cold rotary draw bending by using the analytical modeling, the explicit/implicit 3D-FE modeling, and experiments, indicating that the angular springback increases linearly with increasing the bending angle, while the radius growth slightly fluctuates with the increase of bending angle at the latter bending stage [16]. Zhang et al. constructed a finite element (FE) model for heating-bending of a large-diameter pure titanium tube [6]. From the view of material property, the influences of anisotropy, the Bauschinger effect, and the degradation effect of elastic modulus on springback of tube bending were also studied [17,18,19,20]. For the springback in warm forming of titanium alloys, Ozturk et al. experimentally studied the influence of temperature on springback in bending of a commercial pure titanium (CP-Ti) sheet from room temperature to 300 °C, and revealed that springback is dramatically reduced at elevated temperatures [21]. Zong et al. studied springback characteristics in the V-bending process of a Ti-6Al-4V sheet in the hot forming temperature domain, in which the negative springback phenomenon was found at 850 °C [22]. Grèze et al. studied the springback behavior in an aluminum alloy at different temperatures by using both experimental and numerical approaches [23]. In this study, a split-ring test is used to calibrate the springback amount and revealed that the effect of temperature tends to reduce the stress gradient in the cup wall that is directly linked to the decrease of the springback opening of the ring. Furthermore, Simões et al. numerically studied the influence of the clearance between the die and the punch on springback using the split-ring test [24]. Saito et al. examined the elastoplastic behavior of 980 MPA nano-precipitation strengthened steel at elevated temperatures ranging from room temperature to 700 °C, and further used FE simulation to clarify the effect of stress relaxation and unloading creep on springback in warm V- and U-bending processes [25].

The above studies provide a good knowledge basis for springback analysis. However, tube warm bending-related springback is less reported. Especially for the local-heat-assisted rotary draw bending of thin-walled titanium tubes, both the non-uniform local thermal effect and multi-tool boundaries jointly create a challenge to springback analysis. This paper takes the local-heat-assisted bending of a thin-walled CP-Ti tube as a case, attempting to clarify the springback characteristics through the coupled thermal-mechanical modeling approach. Based on the model, the evolutions of residual stress distribution will be revealed, and influences of temperature and bending angle on springback will be identified to provide a fundamental understanding of the springback phenomena in warm bending of titanium tubular materials.

## 2. Analysis of Local-Heat-Assisted Ti-Tube Bending Processes

**Figure 1:**
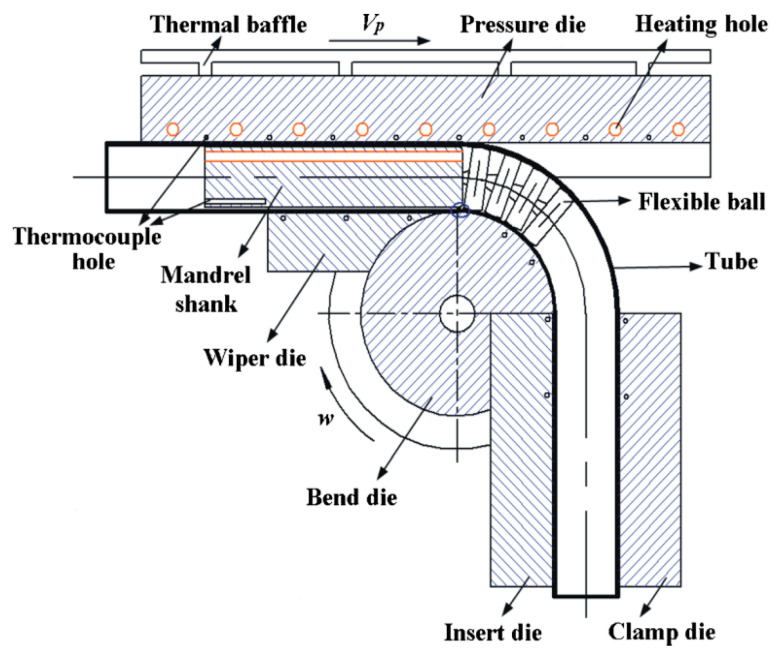
Schematic of local heat-assisted bending of thin-walled titanium tube, adapted with permission from Springer Nature [6].

Local-heat-assisted tube bending is developed by introducing the local thermal field to the conventional rotary draw bending process, thus improving the bending formability and forming quality for hard-to-deform tubular materials. Figure 1 shows the assembly schematic of warm bending of a large-diameter thin-walled titanium tube. Similarly, the tool system includes pressure die, wiper die, clamp die, bending die, insert die, and mandrel die with several flexible balls. The tube is clamped by the clamp die and the insert die connecting to the bending die. By rotating the machine arm, the tube can be drawn to rotate along with the bending die, which makes the tube bend to a certain curvature structure. At the same time, the pressure die moves forward to provide a push-assistant force to feed material. The wiper die and mandrel die provide support to avoid wrinkling and over-flattening defects during the bending process.

In the rotary draw bending process, the bending deformation mainly occurs at the tube behind the clamping zone, which is a certain zone of the tube between pressure die and wiper die. Thus, only this zone needs to be heated to the pre-designed temperature distribution. For the clamping zone, there is no need to heat it since it should be maintained at a low temperature to provide enough stiffness for clamping. As shown in Figure 1, the temperature distribution of the tube is realized by heat transfer from the heated pressure die and mandrel die. Electrical resistance elements that are inserted into the holes are used for heating. A series of thermocouples are applied for real-time monitoring of temperature at different positions, and a temperature control system is developed to automatically adjust the multi-point heating power, thus ensuring the targeted forming temperature field for thin-walled titanium tube bending. To avoid the over-heat transfer to the machine system, the thermal baffle with ribs and water-cooling pipes is applied. Although the multi-point accurate temperature can be sustained by the local heating method, it also leads to the non-uniform temperature distribution of bending tools due to the transferring of complex heat.

**Figure 2:**
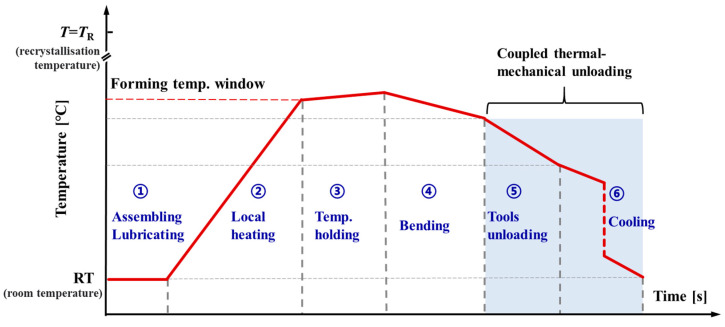
Thermal-mechanical solution for local heat-assisted bending.

As shown in Figure 2, the whole warm bending process can be divided into six stages, viz., assembly and lubricating, local heating, temperature holding, warm bending, tools unloading, and natural cooling. The local heating and holding stages ensure the thermal field within the forming temperature window. Then, under the coupled action of non-uniform temperature field and multi-tool constraints, continuous local elastic-plastic deformation is accumulated for bending the tube to a targeted angle. Finally, the tools are removed and the bent tubular part cools down. In cold forming, springback generally only occurs at the instant of removing tools. However, for warm or hot forming, the non-uniform residual stresses may also create change during the cooling process, which would induce a dimensional variation. Therefore, the unloading springback in warm bending is a coupled thermal-mechanical process. Here, we name the springback in warm bending as two components, transient springback, and cooling springback. In this study, the CP-Ti, Grade 3 tubes with a nominal dimension of 76.2 mm × 1.07 mm (outer diameter × wall thickness) are used as case material in bending.

## 3. Springback Modeling for Tube Warm Bending

Unloading springback is normally a “final step” in a forming operation so that it is sensitive to the through-process history. In local-heat-assisted tube bending, coupled thermal-mechanical springback is crucially affected by the heating, warm bending as well as unloading processes. Thus, from the view of the FE simulation, it is necessary to realize the thermal-mechanical coupled modeling of the whole above-mentioned process. In the following sections, the thermal-mechanical FE model of heating, bending, and unloading, based on the Abaqus, version 2016, will be presented and the experimental details will be introduced.

### 3.1. Coupled Thermal-Mechanical Modeling

**Figure 3:**
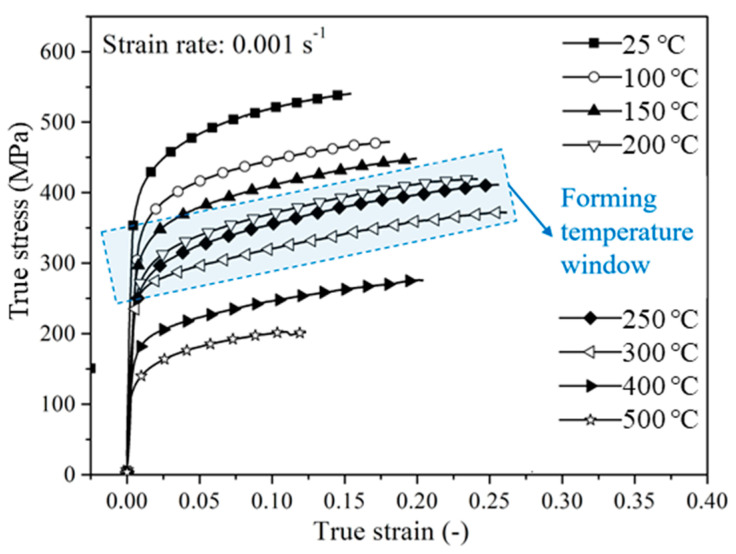
True stress-strain curves of CP-Ti tube at various temperatures.

Considering the characteristics of multi-tool constraints and non-uniform temperature field in local-heat-assisted bending of thin-walled titanium tube, a thermal-mechanical 3D-FE model of heating-bending-unloading is composed of three sub-models, viz., heating and heat transfer model, coupled thermal-mechanical bending model and coupled thermal-mechanical springback model. The three sub-models are sequentially coupled. The heating and heat transfer model is based on the implicit algorithm and considers the full-size geometries of tools and tube blank to ensure the simulation accuracy of temperature distribution. Due to the multi-tool constraints that induce complex contacts in rotary draw bending, the implicit solver can hardly get convergence for solving such kinds of problems. Thus, the warm bending model is based on the dynamic explicit algorithm, in which the non-uniform temperature is passed from the heating/transfer simulation. The tube is discretized by the coupled temperature-displacement shell elements S4RT with reduced integration and hourglass control. The total number of elements is 19,159, in which the element size for the middle bending zone is 2 mm × 2.4 mm (longitudinal × hoop), and the element size for the clamping zone and end-straight zone is 3 mm × 2.4 mm. To improve computation efficiency, the rigid body is used to model the tool geometry and the symmetric semi-tube modeling method is applied. The details such as physical properties, meshing, etc., involved in the FE modeling of heating and warm bending can be found in [6]. Here, only the material properties of CP-Ti tubular material are introduced. According to [6], the true stress-strain curves of the uniform deformation stage (before necking) of the CP-Ti tube are at 25~500 °C, as shown in Figure 3. It could be found from this figure that the CP-Ti present a significant improvement of the elongation and the uniform deformation under 150~300 °C, which could be possibly used as the temperature window for bending process. By using the Swift hardening equation, $\sigma =K{\left(\varepsilon +b\right)}^{n}$, strain-stress curves in the uniform deformation stages are fitted to obtain the basic parameters, viz., elastic modulus (*E*), strength coefficient (*K*), hardening exponent (*n*), normal anisotropy exponent (*r*), and prestrain coefficient (*b*), as shown in Table 1. 

In consideration of the pronounced normal anisotropy of the CP-3 tube, the Hill48 yield function [26], with the isotropic hardening law fitted by the above described Swift equation, is used to describe the normally anisotropic deformation in the FE simulation of the warm bending process. The Hill yield function can be written as:

(1) $$f\left(\sigma \right)=F{\left(\sigma_{22}-\sigma_{33}\right)}^{2}+G{\left(\sigma_{33}-\sigma_{11}\right)}^{2}+H{\left(\sigma_{11}-\sigma_{22}\right)}^{2}+2L\sigma_{23}^{2}+2M\sigma_{31}^{2}+2N\sigma_{12}^{2}-{\overset{¯}{\sigma }}^{2}=0$$

where subscripts 1, 2, and 3 refer to the principle anisotropic axes. *F*, *G*, *H*, *L*, *M*, and *N* are the anisotropic coefficients, which can be calibrated by the uniaxial tension tests at 0°, 45° and 90° with respect to the longitudinal direction of the tube. Under the plane-stress condition, the Hill’48 model depends only on the four coefficients *F*, *G*, *H*, and *N*.

Various methods could be used to calibrate the coefficients of the Hill’48 plasticity model, including the yield stress-based method, *r*-value based method, and combined approach using yield stress and *r*-values [27]. In the present paper, the *r*-value based calibration method was employed to identify the coefficients. It should be noted that tube bending is a process dominated by axial deformation, as well as the difficulty in experimental testing of mechanical properties in the hoop direction of the tube, the in-plan property was reasonably assumed. Consequently, the model parameters in the plane-stress state were finally determined by using the *r*-values obtained at different temperatures, as shown in Table 1.

**Figure 4:**
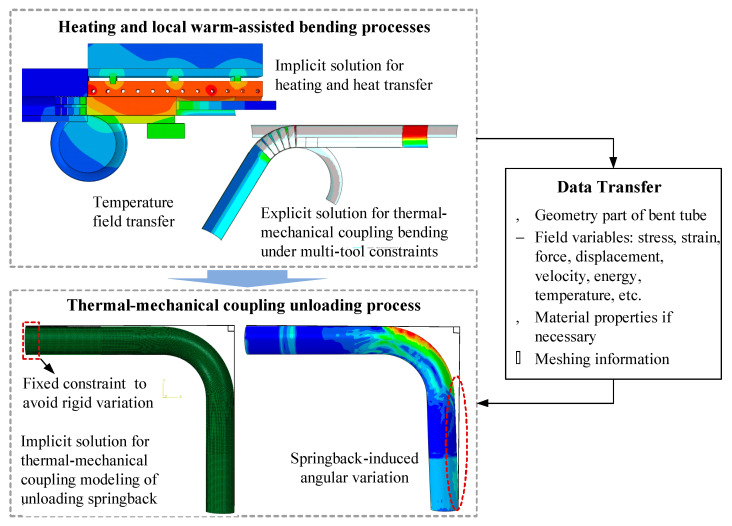
Flowchart for coupled thermal-mechanical modeling of springback.

In the unloading stage, springback occurs when the clamp die is removed from the bent tube. During the process of removing the clamp die, the pressure die is still maintained. After the transient springback is completed, the pressure die is removed. In the transient unloading process, the residual stresses of the bent tube are relieved under a constraint at the end-straight zone. Thus, as shown in Figure 4, to simplify the unloading process in FE modeling, only the bent tube is reserved. A fixed constraint is applied at the end-straight zone of the tube. The bent tube part can be imported into the springback model by “File → Import → Part” from the ODB file of the bending simulation. As the bent tube in the springback model inherits the field information, viz., stress field, strain field, temperature field, etc., from the bent tube after warm bending deformation, all these state variables should be transferred from the warm bending model to the springback model. To realize this purpose, the pre-defined field is used to define the field information of the bent tube after bending. Springback is an elastically-driven deformation process so that only the elastic properties of materials should be defined. If the material properties are not re-defined, the default elastic properties upon unloading are the same as those in the deformation stage. However, some studies have reported that the elastic modulus of titanium upon unloading after a certain level of plastic deformation is less than the modulus in the initial loading stage [28,29,30]. To improve the springback simulation accuracy, the modulus degradation effect can be considered by several nonlinear elastic unloading models in springback simulation for improved prediction accuracy [31,32,33]. This work mainly focuses on the mechanical and thermal issues related to springback characteristics during unloading. The elastic properties used in unloading are considered the same as the constant elastic modulus in the loading stage.

To simulate the thermal-mechanical springback behavior, the “Coupled Temp-Displacement (Transient)” analysis step is employed. Due to the significant springback of the titanium tube, “Nlgeom” is set as “On” to accommodate the nonlinearity in displacement and geometry. In order to control the model stability, the dissipated energy fraction is defaulted by the system and the specific damping factor of 0.0002 (default value) is used to guarantee the convergence. According to the experiment, the time period is set as 1500. The initial increment size is 0.01 and the maximum increment size is 100, while the minimum increment size is 10^−9^ and the maximum increment step number is 1. Since only the bent tube part is in the FE model of springback, it is not necessary to consider the contact friction. As mentioned above, only the end-straight zone of the formed tube is fixed by the pressure die during unloading. Thus, a fixed constraint is set to avoid the rigid displacement in springback modeling. As shown in Figure 4, the freedom degree of translational direction and rotation direction should be released to ensure free deformation. In addition, due to the half-tube model that is used, the asymmetric boundary is imposed on the tube symmetric surface.

### 3.2. Model Verification

Using the established thermal-mechanical-coupling FE model, the whole-process simulation of heating-bending-unloading for warm rotary draw bending of the CP-Ti thin-walled tube is carried out. To verify the established springback model, the ratio of pseudo strain energy (ALLAE) to internal energy (ALLIE) of the bending model is first assessed and is far less than 10%, indicating that the hourglass effect is very slight and can thus be ignored. From the theoretical view, the coupled model is reliable. Furthermore, typical bending experiments are conducted at four sets of forming temperature conditions, viz., 150 °C, 200 °C, 250 °C, and 300 °C, to evaluate the simulation accuracy of springback. The bending radius is *R* = 152.4 mm (*R* = 2*D*), and the bending angle is 90°.

**Figure 5:**
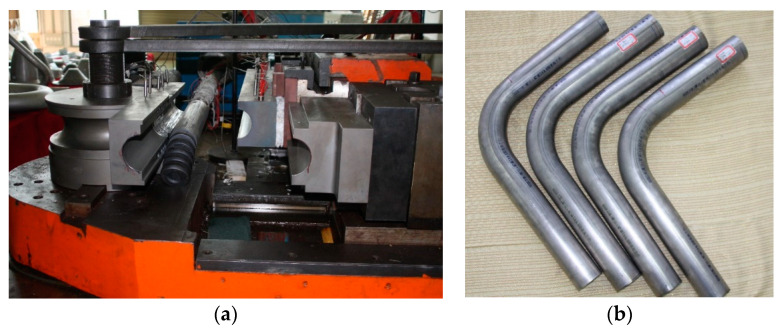
Warm bending experiment of CP-Ti tubes: (**a**) experimental platform; (**b**) typical experimental bent tubes.

Figure 5 illustrates the experimental platform for warm bending and the experimentally formed tubes. It could be found that the overall trend of springback prediction well meets the experiments. However, the prediction error of the springback angle is still at a high level. The reason for the error can be attributed to that the constitutive models used in bending and unloading can hardly accurately characterize the elastic-plastic behaviors of the CP-Ti tube. CP-Ti exhibits a certain extent of tension-compression asymmetry, which cannot be captured by the Hill48 yield criterion and isotropic hardening law used in this work [34]. Furthermore, the unloading elastic modulus presents a significant degradation and then trends to be a saturated value with the accumulation of plastic strain [18,28]. The former can result in the changes in stress-strain distribution of the bent tube, and further affects the neutral layer shifting and bending moment. The latter can directly affect the elastic recovery strain, thus making the springback angle in this study smaller. The above two aspects should be carefully considered in future research for more improved springback prediction. 

In this study, to address the simulation error, a corrected approach is proposed based on the typical experiments and FE simulation. First, an average relative error of springback simulation can be constructed as:

(2) $$\bar{e}=\left(\left|\Delta \theta^{\exp}-\Delta \theta^{\mathrm{pred}}\right|/\Delta \theta^{\exp}+\left|\Delta \theta^{\exp}-\Delta \theta^{\mathrm{pred}}\right|/\Delta \theta^{p\mathrm{red}}\right)/2$$

where $\Delta \theta^{\mathrm{pred}}$ and $\Delta \theta^{\exp}$ are the simulative and experimental springback angles, respectively.

Then, a corrected coefficient can be defined as:

(3) $$\alpha =1+\bar{e}$$

Accordingly, the predicted springback angle can be corrected as:

(4) $$\Delta \theta_{\mathrm{corrected}}^{\mathrm{pred}}=\Delta \theta^{\mathrm{pred}}\cdot \alpha$$

Consequently, from Equation (4), the prediction of the springback angle can thus be corrected.

**Figure 6:**
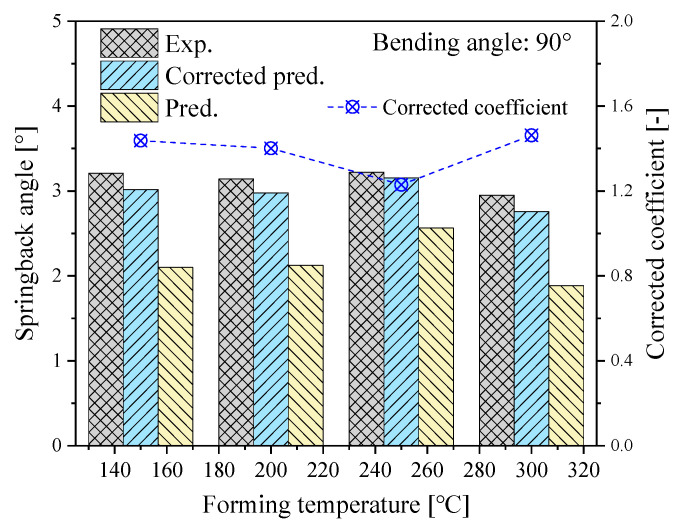
Comparison between predicted and experimental springback angles.

It can be found from Figure 6 that the corrected prediction of springback angles can well agree with the experimental results with a maximum error of less than 6.5%. Li et al. studied the springback rules of titanium tubes at different bending radius and bending angles via experimental, analytical, and numerical simulation approaches, indicating that the analytically and numerically predicted springback angle presents the same trend with the experiments even though there are some differences in the exact values [16]. Thus, a constant corrected coefficient is assumed under different bending angles in the following analysis.

## 4. Results and Discussion

Based on the established FE model, the thermal-mechanical-affected springback characteristics, including time-dependent evolution of springback angle, stress distribution, influences of bending angle on springback, etc., are analyzed and discussed in the following sections.

### 4.1. Springback Characteristics during Transient Unloading and Cooling

#### 4.1.1. Variation of Springback Angle

**Figure 7:**
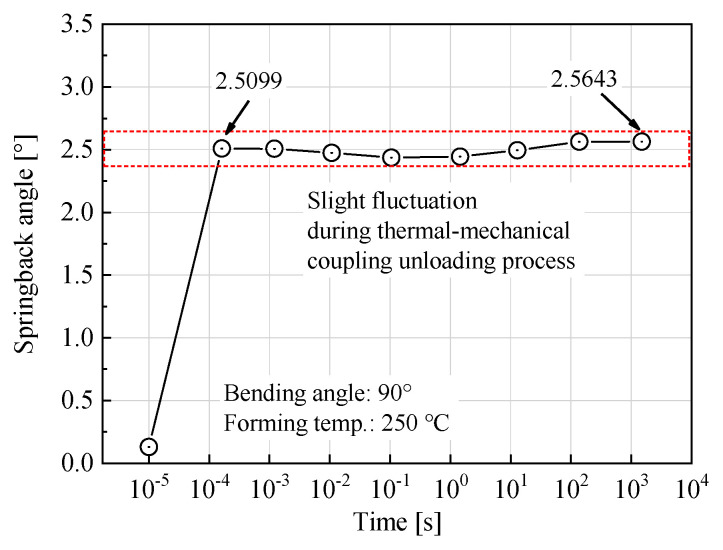
Springback vs. unloading time under 90° bending angle at 250 °C.

During the thermal-mechanical unloading process, the springback angle significantly increases first and then trends to a saturated value with slight fluctuation. Figure 7 shows the change of springback angle with time under 90° bending angle at 250 °C. As the transient springback is completed within a very short time, the logarithm function is used to demonstrate the time coordinate. 

It can be seen that the transient springback is almost completed with 10^−3^ s, and the springback angle is 2.5099°. During the remaining time from 10^−3^ to 1500 s, the springback angle is in a minor fluctuation with a maximum of 0.0544°, which is about 2.2% of the transient springback. The fluctuation of springback during cooling is quite small, which could be possibly covered by the error of measurement. Even so, it is noted that the dynamic evolution of the stress distribution during the cooling process of the bent tube might affect the springback angle also. On the whole, the springback is mainly caused by the transient springback, and the cooling springback could be ignored in analysis.

#### 4.1.2. Evolution of Residual Stress Distribution

**Figure 8:**
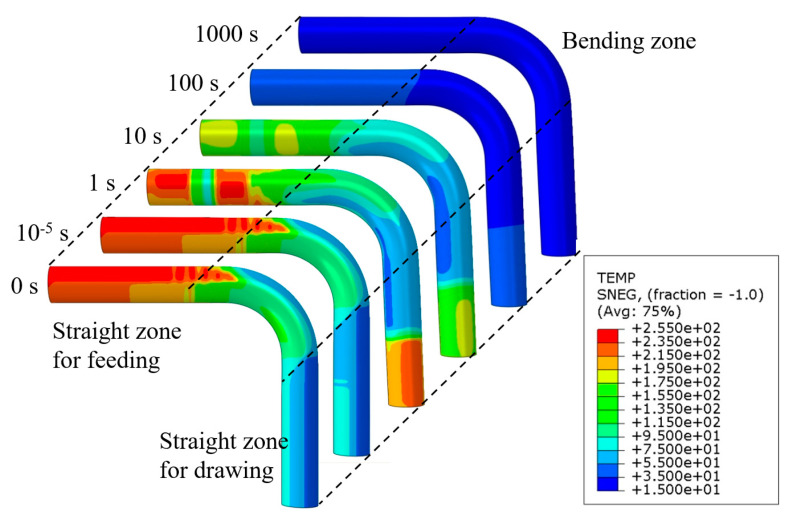
Residual stress distribution of bent tube before unloading under 90° bending at 250 °C.

Springback in tube bending is actually a process where the residual stress of the bent tube is partially released after removing the external tools. Clarifying the stress variation of the bent tube will be helpful to explore the thermal-mechanical-affected springback rules. Figure 8 shows the stress distribution of the bent tube at different unloading moments under 90° bending at 250 °C. The significant change in stress distribution occurs within one second. In this period, the transient springback is completed, and the stress variation is mainly caused by the tool-removal induced elastic unloading. The stress of the straight zone is dramatically decreased. For the bending zone, the stress of extrados that is close to the pressure die is increased, however, the stress of intrados is reduced. As the natural cooling goes on, the residual stress in the area near the pressure die in the extrados is continuously increased, however, there is no distinct change in the stress of the intrados. This phenomenon is caused by non-uniform cooling. As the bent tube cools down, the strength becomes higher. During the cooling process, however, the deformation of this zone is constrained by other portions that have lower temperatures, thus leading to an increase of residual stress.

#### 4.1.3. Evolution of Temperature Distribution

**Figure 9:**
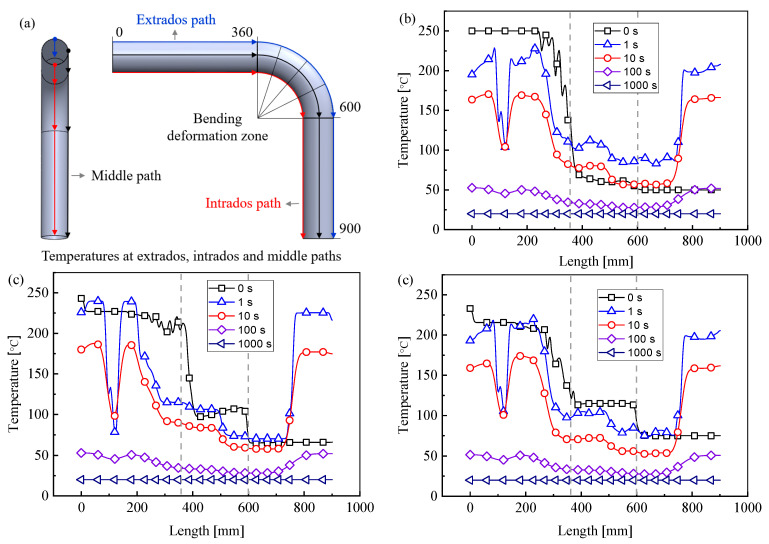
Evolution of temperature field with unloading process under 90° bending at 250 °C forming condition: (**a**) schematic of temperature paths; (**b**) temperatures in the extrados; (**c**) temperatures in the middle; (**d**) temperatures in the intrados.

For the better observation of the change of tube temperature along the axis direction, three paths including the extrados path, middle path, and intrados path are chosen as the reference of observation. Figure 9 shows the temperature variation of the bent tube along the axis direction when the bending temperature is 250 °C and the bending angle is 90°. The bending deformation zone of the tube is shown in the part between the dotted lines. The temperature of the tube in the axis direction changes greatly in the bending deformation zone near the side of the pressure die. This is because the tube is heated by the pressure die and the mandrel before the warm bending process, but it is directly exposed to the air, along with rapid heat dissipation during the warm bending process. It also can be seen that in 10 s, the temperature variation of the zone that is in contact with the mandrel and pressure die is the greatest. This is due to the highest initial temperature in this zone, which leads to a higher rate of heat dissipation.

### 4.2. Forming Temperature-Related Springback Characteristics

#### 4.2.1. Effect of Forming Temperature on Springback

Based on the FE model, the springback rules under different bending angles at different temperatures are obtained. It can be clearly seen from Figure 6 that when the bending temperature is less than 250 °C, the springback angle increases with the increase of temperature and reaches the maximum at 250 °C. After that, the springback angle decreases gradually with the increase of temperature from 250 °C to 300 °C. Within 150~250 °C, the springback angle increases at a slow rate with the increase of temperature. As the temperature increases, the deformation resistance of the CP-Ti is decreased so that the stress level of the bent tube becomes lower, and the material flow becomes easier. In this case, the portion of elastic deformation in the bending deformation zone of the tube may increase, resulting in the variation of springback with temperature. When the temperature ranges from 250 °C to 300 °C, the springback angle decreases slowly with the increase of temperature. The is because the temperature of the bending zone is at a higher level, therefore both the yield strength, flow stress, and elastic modulus of the tube decrease significantly, which may lead to the decreases of the elastic recovery.

#### 4.2.2. Effect of Springback on Tube Thickness at Various Forming Temperatures

During the bending process, the tangential tension strain, hoop tension strain and normal compression strain of the extrados of the bent tube are induced by the tangential tension stress, hoop tension stress, and normal compression stress, which results in the stretching and thinning in the outer side of the bent tube. The tangential compression, hoop tension, and normal tension strains on the inner side of the tube are induced by the tangential compression stress, hoop compression stress, and normal compression stress, which result in the compression and thickening in the inner side of the bent tube, and even lead to the wrinkles. Due to the recovery of the elastic deformation, both the thinning degree at extrados and the thickening degree at intrados would decrease. As the change of thickness due to springback is elastic deformation, the thickness change is very small. Assuming that the elastic deformation of the formed part is completed unloaded, the springback induced change of thickness could be roughly estimated, varying in the range of about 0.12~0.15%. In addition to the elastic deformation induced thickness change, the thermal expansion could also play a role in the variation of thickness. Along with the cooling process, the thickness of the entire tube will decrease. According to SAE [35], the linear thermal expansion coefficient is 8.6 × 10^−6^ m/m/°C within 20~100 °C and 9.5 × 10^−6^ m/m/°C within 20~100 °C. Thus, using the linear thermal expansion coefficient, the thermal expansion induced thickness variation could be roughly estimated as −0.11% within 20~100 °C and −0.27% within 20~300 °C. Thus, by considering the coupled effect of springback and thermal expansion, the change of thickness at extrados during springback would be smaller than that at intrados. Even so, the maximum change of the thinning/thickening degree of the tube thickness is quite minor—less than about 0.4% based on the above estimation, which is much smaller than the thickness changes due to bending deformation and thus could be neglectable for most cases.

#### 4.2.3. Effect of Springback on Tube Cross-Section at Various Forming Temperatures

**Figure 10:**
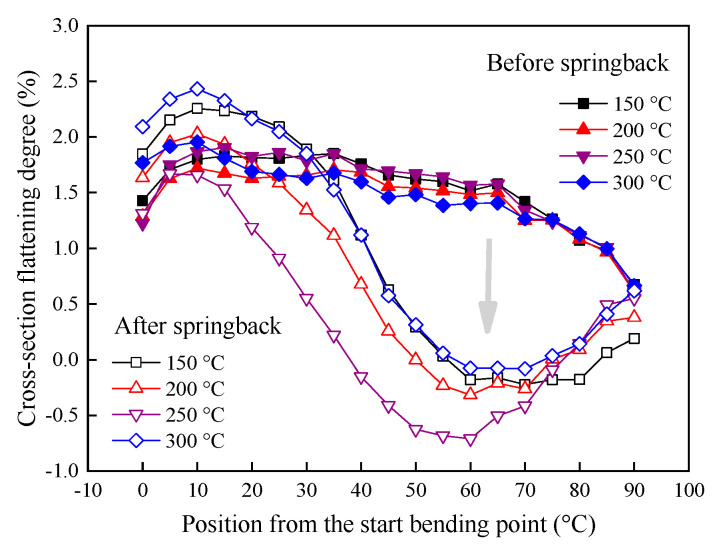
Cross-section flatting degrees under different temperatures.

During tube bending, the tangential tensile stress in the extrados, tangential compressive stress in the intrados, and the total circumferential stress are toward the center of the cross-section of the bent tube under the bending moment, thus making the cross-section to be flattened. In this study, we take the bending case with 90° to analyze the springback induced effect on the cross-section. Figure 10 shows the change of cross-section flattening along bending direction after springback under 90° bending at different temperatures. It can be seen that the maximum cross-section flatting occurs at the bending angle around 10° before and after springback. For the four bending temperatures, the flattening degrees first increase and then gradually decrease along the bending direction before springback. After springback, the flatting degree first increases along the bending direction and then reaches a maximum at the position of 10°, then dramatically decreases and the minimum value occurs at the position of about 60°. Finally, the flattening degree presents a slow increase and gradually approximates to the flattening degree before springback. Therefore, during the unloading springback process, there is a significant change in the overall distribution of flatting degrees after springback.

### 4.3. Bending Angle Related Springback Characteristics

#### 4.3.1. Effect of Bending Angle on Springback

To explore the effects of bending angle on springback, the change of springback angle at the same forming temperature is identified and discussed. Using the bending process at 250 °C as an example for analysis, the change of springback angle under 45°, 90°, and 180° could be calculated as 2.75°, 3.14°, and 3.21°, respectively, indicating that the springback angle increases with the bending angle. It is noted that the increase of the springback angle presents a nonlinearity with the bending angle. The increasing rate of springback tends to decrease at the larger bending angles. This may be caused by the accelerated stress relaxation at elevated temperatures. With the increase of bending angle, the duration time of the whole bending process is accordingly increased. In this condition, the bent zone is held at a high temperature, resulting in the reduction of inner residual stress. Consequently, the increasing rate of springback tends to be slower with the increase of bending angle.

#### 4.3.2. Effect of Springback on Thickness under Various Bending Angles

**Figure 11:**
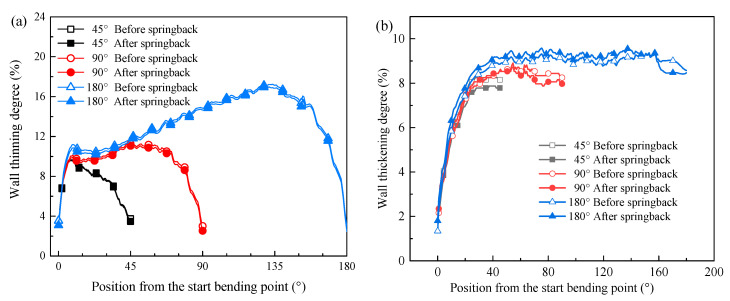
Variation of wall thickness variation under different bending angles at 250 °C: (**a**) thinning of extrados; (**b**) thickening of intrados.

The effects of springback on the wall thickness in the extrados of the bent tube under different bending angles at 250 °C are explored and discussed. As shown in Figure 11a, the larger the bending angle is, the higher the wall thinning degree in the extrados is, and the greater the reduction value of the wall thinning degree in extrados is after springback. Figure 11b shows the evolution of the wall thickening degree in the intrados of the bent tube along the bending direction under different bending angles at 250 °C. It can be seen that the wall thickening degree sharply increases when the angle to the initial bending section is less than 25°, and it is almost unchanged when the angle is greater than 25°. Under the bending angles of 45° and 90°, the wall thickening degree in the intrados of the bent tube reduces after springback, while for the bending condition at 180°, the wall thickening degree slightly increases.

#### 4.3.3. Effect of Springback on Cross-Section under Various Bending Angles 

**Figure 12:**
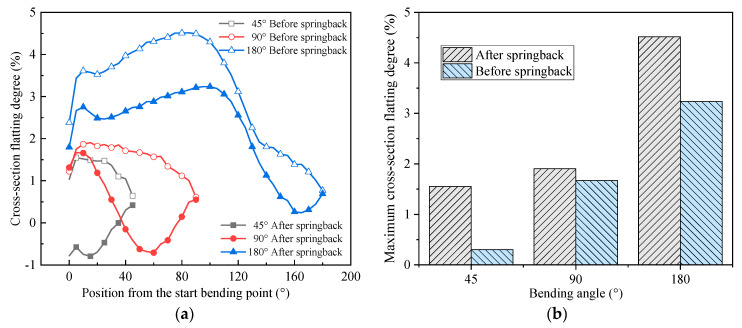
Variation of cross-section flatting under different bending angles at 250 °C: (**a**) distribution along bending angle; (**b**) maximum flattening.

To explore the effects of springback on the cross-section flatting under various bending angles, the simulations at 250 °C are carried out for analysis. Figure 12a shows the change of the cross-section flatting degree along the bending direction before and after springback under different bending angles at 250 °C. It can be seen that the cross-section flatting degrees all decrease after springback under different bending angles. The flatting degrees all first increase and then decrease and finally increase along the bending direction after springback. Cross-section behavior is affected by both bending geometry and tooling. In particular, for the rotary draw bending process in this study, in addition to the clamp die, pressure die, wiper die, and bend die applied on the outside surface of the tube, there is also mandrel die with several flexible balls inside the tube. The mandrel die could significantly affect the flattening behavior during bending. It could be found from the three curves after springback that there is a zone where the flattening degree is negative. This may attribute to extension displacement of the mandrel rod with respect to the bending tangent point. Thus, a local deformation at the end of the mandrel rod could be formed during bending, which results in the negative flattening after springback. It also could be found from Figure 12b, that, the maximum flatting degree increases with bending angle before and after springback, and the maximum flatting degree after springback is less than that before springback; the reduction value first decreases and then increases with the increase of the bending angle.

## 5. Conclusions

Combined with representative experiments and coupled thermal-mechanical FE modeling, the springback characteristics of thin-walled CP-Ti tubes in the local-heat-assisted rotary draw bending process were systematically investigated. The main findings are summarized as follows:

- Springback in local-heat-assisted bending of a CP-Ti tube is a coupled thermal-mechanical process, which can be divided into transient springback, along with removing external tools and cooling-induced springback and natural air cooling. By combining the implicit and explicit solving algorithms, a coupled thermal-mechanical FE model for springback prediction is established and verified.
- Based on simulation and experiment, thermal-mechanical springback characteristics are revealed: springback mainly occurs upon transient unloading stage, and then presents slight fluctuation with a maximum of 2.2% of the total springback during air cooling; under the same bending angle, springback increases initially and then decreases with the increase of the forming temperature; under the same forming temperature, springback angle increases with a degraded rate at higher bending angles.
- Springback induced variations on tube thickness and cross-section flattening are discussed: the thickness variation during springback is attributed to the elastic unloading induced deformation and the cooling induced thermal deformation, which is small and neglectable; the maximum cross-section flattening decreases first and then presents an increasing trend, while it increases with the increase of the bending angle.

## Figures and Tables

**Table 1:** Mechanical properties of CP-Ti tube at various temperatures.

*T* (°C)	Fundamental Material Parameters						Coefficients of Hill’48 Model			
	*E* (GPa)	$\sigma_{0}\;(\mathrm{MPa})$	*K* (MPa)	b	*n*	*r*	*F*	*G*	*H*	*N*
25	82.45	381	650.25	0.0040	0.0970	1.78	0.36	0.36	0.64	3.28
100	73.22	326	557.82	0.0035	0.0950	2.00	0.33	0.33	0.67	3.33
150	66.67	298	546.90	0.0077	0.1247	2.30	0.30	0.30	0.70	3.39
200	66.1	258	523.21	0.0102	0.1543	2.40	0.29	0.29	0.71	3.41
250	64.77	244	513.31	0.0111	0.1658	2.30	0.30	0.30	0.70	3.39
300	61.83	220	481.15	0.0262	0.2146	2.34	0.30	0.30	0.70	3.40

## Data Availability

Not applicable.
